# Knowledge, attitudes and practices toward artificial intelligence among pediatricians in India: A cross-sectional web-based nationwide survey

**DOI:** 10.1371/journal.pone.0338314

**Published:** 2025-12-12

**Authors:** Manasvi Tyagi, Rohit Ravi, Suchetha Sudesh Rao, Nutan Kamath, Santhosh T. Soans

**Affiliations:** 1 Dept. of Pediatrics, Kasturba Medical College Mangalore, Manipal Academy of Higher Education, Manipal, India; 2 Department of Audiology and Speech Language Pathology, Kasturba Medical College Mangalore, Manipal Academy of Higher Education, Manipal, India; 3 Department of Pediatrics, AJ institute of Medical Sciences, Mangalore, Karnataka, India; University of Birmingham, UNITED KINGDOM OF GREAT BRITAIN AND NORTHERN IRELAND

## Abstract

**Background:**

Artificial intelligence (AI) is rapidly advancing in healthcare and has the potential to transform patient care. This study aimed to assess the knowledge, attitudes, and practices (KAP) regarding AI among pediatricians in India.

**Methods:**

A cross-sectional, web-based nationwide survey was conducted, with participants recruited via pediatric WhatsApp groups between 23 March, 2024 to 1 April 2024. A validated 25-item questionnaire, comprising multiple-choice, open-ended, yes/no, and five-point Likert scale questions, was administered using Google Forms. Descriptive statistics were used to summarize responses.

**Results:**

A total of 116 pediatricians participated in the survey. The calculated sample size for the study was 114. As we had 116 responses on day 10, the survey was withdrawn accounting to a response rate of 6.7%. Most participants (91.4%) understood the concept of AI; however, only 33.6% were familiar with AI applications in pediatric practice. The majority (83.6%) anticipated the integration of AI in pediatrics within the next decade, and 90.5% expressed interest in learning more about AI applications. Notably, 25.9% reported concerns regarding AI use, and 48.3% perceived their workplaces as currently not adequately equipped to implement AI tools. Commonly used AI tools included ChatGPT, Canva AI, Neonatal AI, Google AI, Trusted Computing Base (TCB), and Voicera.

**Conclusion:**

While awareness of AI and its potential impact was high, knowledge and formal training were limited. Nevertheless, there was considerable enthusiasm among pediatricians to acquire further knowledge and adopt AI applications in their practice.

## Background

Artificial intelligence (AI) is increasingly transforming healthcare, with applications in diagnostic decision support, medical imaging, drug discovery, surgical assistance, virtual health assistants, and remote patient monitoring [1–3]. AI systems have demonstrated strong performance in tasks such as medical image interpretation, natural language processing for clinical data, speech recognition, semantic reasoning, and predictive analytics, sometimes achieving accuracy comparable to or exceeding that of human clinicians [4–6]. The integration of AI into healthcare has the potential to improve diagnostic accuracy, optimize treatment pathways, and enhance patient outcomes, although concerns regarding training, ethics, and adoption remain among healthcare professionals [7].

Several international surveys have explored physicians’ knowledge, attitudes, and practices (KAP) regarding AI in medicine. These studies, conducted across specialties such as dermatology [8], psychiatry [9], radiology [10,11], pediatrics [12], surgery [13], and among medical students and residents [14–16], generally report limited knowledge and training but a high level of interest in AI’s potential applications. For example, radiologists and psychiatrists have shown optimism toward AI tools but also uncertainty about integration into clinical workflows. Similar trends have been reported among medical students and residents, who express enthusiasm but lack structured educational opportunities.

In India, research on physicians’ perspectives toward AI remains limited, with few studies addressing pediatricians specifically. Pediatric care presents unique challenges such as child-specific diagnostics, developmental monitoring, and the need for family-centered approaches that may shape how pediatricians view and adopt AI. Understanding these perspectives is critical to guide the development of appropriate training programs, policy initiatives, and the inclusion of AI-related content in medical curricula.

Nasscom has predicted that AI and AI data is expected to generate about 30 billion US dollars to the GDP of India. [17] AI based health innovations have significantly impacted Ayushman Bharat Digital Mission. With the industry academia collaboration, AI with algorithms in machine learning and deep learning is contributing to prediction of disease, analysis of medical images, personalized plans for treatment, platforms for telemedicine, AI backed diagnostics and wearable devices in the ecosystem of Indian healthcare. [18].

The primary objective of this study was to evaluate the knowledge, attitudes, and practices of pediatricians in India regarding the use of AI in Pediatric practice. A secondary objective was to explore their interest in education and training in AI and their willingness to integrate AI tools into pediatric practice.

## Materials and methods

The present study received ethical approval from the Institutional Ethical Committee of KMC Mangalore (IEC KMC MLR 03/2024/178). A cross-sectional, web-based survey was carried out to evaluate the knowledge, attitudes, and practices (KAP) regarding AI among pediatricians practicing in India. The online consent was obtained from the participants before participation in the study.

The questionnaire developed by Pecqueux et al. [13] served as the basis for the survey, with modifications to incorporate multiple-choice, open-ended, dichotomous (yes/no), and five-point Likert scale questions. The instrument was subjected to content validation by five pediatricians with over ten years of professional experience, using a four-point relevance scale. Items rated as “relevant” or “quite relevant” were retained following the calculation of the scale content validity index (S-CVI) [19]. The resulting S-CVI of 0.88 indicated excellent content validity.

The finalized questionnaire consisted of 25 items: 6 demographic questions, 9 assessing knowledge, 6 evaluating attitudes, and 4 addressing practices related to AI. This version was converted into an online format using Google Forms, with all questions made mandatory to ensure completeness of responses. Data collection was conducted between 23 March 2024 and 1 April 2024. The survey link was disseminated via WhatsApp to 1,725 pediatricians across India to ensure pan India representation of practicing Pediatricians with minimum two years’ experience in the field. Retired Pediatricians and academic residents were excluded from participation in the study. Distribution channels included the Indian Academy of Pediatrics Dakshina Kannada branch (185 members), the Pediatric Rheumatology Group (129 members), Kasturba Medical College Pediatric Alumni Group (325 members), the Mahatma Gandhi Institute of Medical Sciences Pediatric Alumni Group (114 members), the National Women’s Pediatricians Forum (947 members), and the authors’ personal contacts (25 members). The survey remained open till we achieved the calculated sample size (10 days) A brief introduction outlining study objectives and confidentiality assurances was provided, and informed consent was obtained prior to participation. The estimated completion time was 8–10 minutes. All responses were reviewed by the principal investigator.

The sample size was calculated for estimation of population proportion based on the previous study using the formula: n = x^2^pq/e^2^. At 95% confidence level and with 5% level of significance, z-score (z) is 1.96. Margin of error (e) = 4%, Population proportion p = 0.95 and q = 0.15 [20] Substituting values to the above formula, the sample size (n)= 114.

The study adhered to the Checklist for Reporting Results of Internet E-Surveys (CHERRIES) guidelines [21]. Descriptive statistics were computed using SPSS Version 29 (IBM Corporation, Armonk, NY, USA), with means, standard deviations, and ranges calculated for continuous variables, and frequencies and percentages reported for categorical variables.

### Results

A total of 116 pediatricians completed the survey as per our sample size calculation, yielding a response rate of 6.7%. Among the respondents, 45 (38.8%) were female and 71 (61.2%) were male. Participant ages ranged from 27 to 82 years, with a mean of 46 years (±13.04). The demographic characteristics of the participants are summarized in Table 1.

**Table 1:** Demographic details of participants.

		Frequency (Percentage)
**Experience (in years)**	0-5 years	20 (17.2%)
	6-10 years	20 (17.2%)
	11-15 years	10 (8.1%)
	16- 59 years	58 (50%)
	>60 years	8 (7.5%)
**Education**	MD/DNB	100 (86.2%)
	DM/DrNB	16 (13.8%)
**Practice Location**	City	38 (32.8%)
	Metropolitan	52 (44.8%)
	Small town	26 (22.4%).
**Practice Set-up**	Hospital setup	41 (35.3%)
	Medical college-hospital setup	44 (37.9%)
	Private practice	31 (26.7%)

The responses to the Yes, No and Not sure questions exploring knowledge, attitude and practice towards AI tool are tabulated in Table 2a, 2b and 2c.

**Table 2:** a: Knowledge towards AI tool. b: Attitude towards AI tool. c: Practice towards AI tool.

a			
**Knowledge**	**Yes**	**No**	**Not sure**
Do you understand the meaning of AI?	106 (91.4%)	0	10 (9.6%)
Do you know the difference between AI, deep learning, neural networks and machine learning?	47 (40.5%)	39 (33.6%)	30 (25.9%)
Have you received any formal training in AI?	1 (0.9%)	115 (99.1%)	0
Are you familiar with applications of AI in pediatric practice?	39 (33.6%)	60 (51.7%)	17 (14.7%)
Do you think that AI will be used in pediatric practice in the next 10 years?	97 (83.6%)	05 (4.3%)	14 (12.1%)
**b**			
**Attitude**	**Yes**	**No**	**Not sure**
Do you think that your workplace/clinic is adequately equipped for the introduction of AI?	33 (28.4%)	56 (48.3%)	27 (23.3%)
Should there be a better information availability at your workplace/clinic (e.g., training, courses) about the possible future application of AI in pediatric practice?	90 (77.6%)	9 (7.8%)	17 (14.7%)
Would you consider using the following clinical workflow? The clinical images of a patient are analyzed with AI. A specialist (professional experience > 15 years) reviews both- the images, and the AI results and makes a diagnosis based on them.	89 (76.7%)	6 (5.2%)	21 (18.1%)
**c**			
**Practice**	**Yes**	**No**	**Not sure**
Are you currently using any AI tool for application in your practice?	11 (9.5%)	99 (85.3%)	6 (5.2%)

The responses to various multiple-choice questions with different options for exploring knowledge, attitudes and practices are tabulated in Table 3a, 3b and 3c.

**Table 3:** a: Knowledge towards AI tool (Multiple choice). b: Attitude towards AI tool (Multiple choice). c: Practice towards AI tool (Multiple choice).

a		
Knowledge	Options	Frequency & Percentage
How would you rate your knowledge of AI in general	Rudimentary	44 (37.9%)
	Above average	17 (14.7%)
	Average	49 (42.2%)
	No Knowledge	6 (5.2%)
In which area do you see the use of AI? (multiple answers possible)	AI- based clinical assessment (E.g.- Automated image-based bilirubin analysis, AI- assisted detection of murmurs, etc.)	65 (56%)
	AI-based analysis of medical imaging	83 (71.6%)
	AI-based comprehensive analysis of multiple information for diagnostics	76 (65.5%)
	AI- based therapy decisions and pediatric dose calibration	59 (50.9%)
	Others	8 (6.9%)
Which area of healthcare do you think will be the first to use AI commercially? (multiple answers possible)	Public primary care such as public health centers	22 (19%)
	Primary care in private clinics	25 (21%)
	Specialized clinics	57 (49.1%)
	University Hospitals	67 (57.8%)
	Others	11 (9.5%)
b		
Attitude	Options	Frequency & Percentage
How do you feel about the incorporation of AI into pediatric practice?	Very concerned	5 (4.3%)
	Concerned	25 (21.6%)
	Neutral	36 (31%)
	Excited	43 (37.1%)
	Very Excited	7 (6%)
Are you interested in learning more about AI?	Very interested	43 (37.1%)
	Interested	62 (53.4%)
	Neutral	9 (7.8%)
	Little interest	2 (1.7%)
	No interest	0
In your opinion, what level of error is acceptable for AI systems applied for the purpose of diagnosis and treatment of diseases in pediatric practice?	Equivalent to the average performance of an assistant physician (professional experience < 5 years)	35 (30.2%)
	Equivalent to the average performance of a medical specialist (professional experience approx. 5–10 years)	15 (12.9%)
	Equivalent to the average performance of a senior physician (professional experience approx. 10–15 years)	13 (11.2%)
	Equivalent to the average performance of a proven specialist (professional experience > 15 years)	24 (20.7%)
	Superior to the average performance of a proven specialist (professional experience > 15 years)	29 (25%)
**c**		
Practice	Options	Frequency & Percentage
For which of the following do you see the greatest potential disadvantage in using AI systems in your field? (Multiple answers possible)	It is not flexible enough to be applied to every patient	53 (45.7%)
	It is difficult to apply it to controversial issues	43 (37.1%)
	Poor acceptance by the patients	29 (25%)
	Poor ability to empathize and consider the patient’s and parent’s emotional well-being	74 (63.8%)
	It is developed by a specialist with little clinical experience in medical practice	43 (37.1%)
	If complications occur, there are ethical and legal problems regarding liability	85 (73.3%)

*Multiple choice responses, hence, proportions don’t add up to 100%

**Fig 1:**
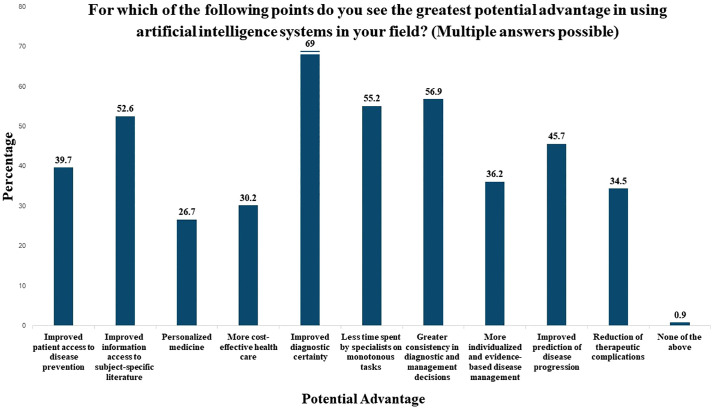
Potential advantage of the use of AI in the field of pediatric practice.

A total of 90.5% of the participants responded that they were very interested/interested in learning more about AI. Overall, there was poor knowledge about AI among the participants, and the participants exhibited positive attitudes towards learning more about AI and its application. However, 73% were concerned about possible ethical and legal problems, which could arise from the use of AI. As depicted in Figure 1, respondents identified several key potential advantages of AI in pediatric practice.

Sixty-nine percent of the participants reported improved diagnostic certainty, 56.9% greater consistency in diagnostic and management decisions, and 55.2% reported less time spent by specialists on monotonous tasks as a potential advantage of AI in pediatric practice. For the open-ended question, which explored the tool used by pediatricians, 99 (85.3%) participants did not use AI tools. ChatGPT was the most preferred tool, and the other AI tools reported were Canva AI, Neonatal AI, Google AI, Trusted compute base (TCB), and Voicera. Overall, the results suggest acceptance of the use of AI among pediatricians, but at the same time, they expressed a need for additional information and training.

### Discussion

This study provides insight into the knowledge, attitudes, and practices (KAP) regarding AI among pediatricians in India, highlighting both opportunities and challenges in adopting AI in pediatric care. Overall, while most respondents (91.4%) understood the term AI, a majority (59.5%) were unaware of AI subtypes, and only one respondent had prior formal training. Despite this limited formal knowledge, most pediatricians (76%) were willing to use AI alongside their clinical expertise, and nearly all (99%) believed that AI applications could positively impact pediatric healthcare. These findings suggest a high degree of optimism and readiness to learn, consistent with international studies showing similar enthusiasm among physicians and pediatricians in Europe, Russia, and Pakistan [8,10,12,14,15,22,23].

Interestingly, while respondents recognized the theoretical potential of AI in improving diagnostic accuracy, streamlining workflows, and enhancing patient care, the tools they most commonly used were general-purpose applications, such as ChatGPT and Canva AI, rather than pediatric-specific AI solutions. This gap between theoretical knowledge and practical usage emphasizes the need for targeted educational programs that provide pediatricians with training in specialty-specific AI tools and applications. The Indian Association of Pediatrics should take initiatives for CME programs to bridge this gap.

Concerns regarding ethical, legal, and practical aspects of AI were also reported. Approximately three-quarters of respondents worried about potential complications, and over 60% expressed concerns about AI’s inability to empathize or consider the emotional wellbeing of children and caregivers. Other concerns included lack of flexibility, difficulties with controversial cases, and reliability of AI applications developed without sufficient clinical expertise. These findings align with previous studies among Russian and English physicians, highlighting shared apprehensions regarding the integration of AI in clinical practice [15,23].

The study also suggests differences in attitudes based on experience and self-reported knowledge, with younger and more digitally literate pediatricians appearing more open to adopting AI. Although subgroup analyses were limited by sample size, these trends underscore the importance of stratified educational strategies to address knowledge gaps across career stages.

Several limitations must be acknowledged. First, the survey had a low response rate (6.7%), which may limit the generalizability of the findings. Second, distribution via WhatsApp may have introduced selection bias toward pediatricians with higher digital literacy. Third, knowledge and attitudes were self-reported, which may not accurately reflect objective understanding or competence in AI usage. Despite these limitations, the study provides valuable preliminary insights into pediatricians’ perceptions of AI in India.

#### Implications and future directions.

The findings highlight the need for structured training and continuing medical education programs on AI in pediatrics. Professional associations and academic institutions can develop workshops, online modules, and curricula that focus on the ethical, clinical, and practical aspects of AI use in pediatric care. Additionally, future research should aim for larger, nationally representative samples, objectively assess AI competence, and evaluate the impact of AI integration on clinical outcomes in pediatric practice.

### Conclusion

Most pediatricians in India are aware of AI and its potential impact on healthcare. However, formal knowledge and training in AI remain limited, and only a small proportion currently use AI tools in clinical practice. While respondents expressed optimism about AI’s ability to support diagnosis, management, and data analysis, many also raised concerns regarding ethical, legal, and practical implications.

These findings highlight an urgent need for structured educational initiatives to improve awareness, knowledge, and responsible adoption of AI in pediatric practice. National professional bodies, such as the Indian Academy of Pediatrics, should lead efforts to provide continuing medical education programs and workshops for practicing pediatricians. Furthermore, integrating AI principles and applications into undergraduate and postgraduate medical curricula is essential to ensure ethically informed and clinically competent use of AI in pediatric healthcare. By addressing these gaps, pediatricians can be better prepared to leverage AI effectively, ultimately improving patient care and outcomes.

## References

[1] McCarthy J, Minsky ML, Rochester N, Shannon CE. A proposal for the dartmouth summer research project on artificial intelligence. AI Magazine. 2006;27(4):12.

[2] TURING AM. I.—Computing machinery and intelligence. Mind. 1950;LIX(236):433–60. doi: 10.1093/mind/lix.236.433

[3] Liu PR, Lu L, Zhang JY, Huo TT, Liu SX, Ye ZW. Application of Artificial Intelligence in Medicine: An Overview. Curr Med Sci. 2021 Dec; 41(6):1105–15. 34874486 10.1007/s11596-021-2474-3 PMC8648557

[4] Bajwa J, Munir U, Nori A, Williams B. Artificial intelligence in healthcare: transforming the practice of medicine. Future Healthc J. 2021;8(2):e188–94. doi: 10.7861/fhj.2021-0095 34286183 PMC8285156

[5] Shu L-Q, Sun Y-K, Tan L-H, Shu Q, Chang AC. Application of artificial intelligence in pediatrics: past, present and future. World J Pediatr. 2019;15(2):105–8. doi: 10.1007/s12519-019-00255-1 30997653

[6] Andykarayalar R, Mohan Surapaneni K. ChatGPT in Pediatrics: Unraveling its Significance as a Clinical Decision Support Tool. Indian Pediatr. 2024;61(4):357–8. doi: 10.1007/s13312-024-3159-3 38450533

[7] Chen M, Zhang B, Cai Z, Seery S, Gonzalez MJ, Ali NM, et al. Acceptance of clinical artificial intelligence among physicians and medical students: A systematic review with cross-sectional survey. Front Med (Lausanne). 2022;9:990604. doi: 10.3389/fmed.2022.990604 36117979 PMC9472134

[8] Polesie S, Gillstedt M, Kittler H, Lallas A, Tschandl P, Zalaudek I, et al. Attitudes towards artificial intelligence within dermatology: an international online survey. Br J Dermatol. 2020;183(1):159–61. doi: 10.1111/bjd.18875 31953854

[9] Doraiswamy PM, Blease C, Bodner K. Artificial intelligence and the future of psychiatry: Insights from a global physician survey. Artif Intell Med. 2020;102:101753. doi: 10.1016/j.artmed.2019.101753 31980092

[10] Coppola F, Faggioni L, Regge D, Giovagnoni A, Golfieri R, Bibbolino C, et al. Artificial intelligence: radiologists’ expectations and opinions gleaned from a nationwide online survey. Radiol Med. 2021;126(1):63–71. doi: 10.1007/s11547-020-01205-y 32350797

[11] Ooi SKG, Makmur A, Soon AYQ, Fook-Chong S, Liew C, Sia SY, et al. Attitudes toward artificial intelligence in radiology with learner needs assessment within radiology residency programmes: a national multi-programme survey. Singapore Med J. 2021;62(3):126–34. doi: 10.11622/smedj.2019141 31680181 PMC8027147

[12] Perrier E, Rifai M, Terzic A, Dubois C, Cohen JF. Knowledge, attitudes, and practices toward artificial intelligence among young pediatricians: A nationwide survey in France. Front Pediatr. 2022;10:1065957. doi: 10.3389/fped.2022.1065957 36619510 PMC9816325

[13] Pecqueux M, Riediger C, Distler M, Oehme F, Bork U, Kolbinger FR, et al. The use and future perspective of artificial intelligence - a survey among German surgeons. Frontiers in Public Health. 2022;10:982335. doi: 10.3389/fpubh.2022.982335 36276381 PMC9580562

[14] Kansal R, Bawa A, Bansal A, Trehan S, Goyal K, Goyal N. Differences in knowledge and perspectives on the usage of artificial intelligence among doctors and medical students of a developing country: a cross-sectional study. Cureus. 2022;14(1). 10.7759/cureus.21434 PMC8860704 35223222

[15] Ahmed Z, Bhinder KK, Tariq A, Tahir MJ, Mehmood Q, Tabassum MS, et al. Knowledge, attitude, and practice of artificial intelligence among doctors and medical students in Pakistan: A cross-sectional online survey. Ann Med Surg (Lond). 2022;76:103493. 35308436 10.1016/j.amsu.2022.103493 PMC8928127

[16] Orlova IA, Akopyan ZA, Plisyuk AG, Tarasova EV, Borisov EN, Dolgushin GO, et al. Opinion research among Russian Physicians on the application of technologies using artificial intelligence in the field of medicine and health care. BMC Health Serv Res. 2023;23(1):749. doi: 10.1186/s12913-023-09493-6 37442981 PMC10339534

[17] NASSCOM. How AI is transforming the future of healthcare in India. https://nasscom.in/knowledge-center/publications/how-ai-transforming-future-healthcare-india. 2023. 2025 November 17.

[18] Kapoor N, Sanjana SN, Davalagi SB, Balu PS, Sethia S. AI Horizons in Indian Healthcare: A Vision for Transformation and Equity. Indian J Community Med. 2024;49(Suppl 2):S210–6. doi: 10.4103/ijcm.ijcm_806_24 40124859 PMC11927818

[19] Polit DF, Beck CT. The content validity index: are you sure you know what’s being reported? Critique and recommendations. Res Nurs Health. 2006;29(5):489–97. doi: 10.1002/nur.20147 16977646

[20] Ravi R, Gunjawate DR, Yerraguntla K, Lewis LE, Rajashekhar B. A national survey of knowledge, attitude and practices among pediatricians towards newborn hearing screening in India. Int J Pediatr Otorhinolaryngol. 2017;95:9–14. doi: 10.1016/j.ijporl.2017.01.032 28576542

[21] Eysenbach G. Improving the Quality of Web Surveys: The Checklist for Reporting Results of Internet E-Surveys (CHERRIES). J Med Internet Res. 2004;6(3). 10.2196/jmir.6.3.e34 PMC1550605 15471760

[22] Hoodbhoy Z, Masroor Jeelani S, Aziz A, Habib MI, Iqbal B, Akmal W, et al. Machine Learning for Child and Adolescent Health: A Systematic Review. Pediatrics. 2021;147(1):e2020011833. doi: 10.1542/peds.2020-011833 33323492

[23] Keane PA, Topol EJ. AI-facilitated health care requires education of clinicians. Lancet. 2021;397(10281):1254. doi: 10.1016/S0140-6736(21)00722-4 33812482

